# Human platelets release amyloid peptides β_1-40_ and β_1-42_ in response to hemostatic, immune, and hypoxic stimuli

**DOI:** 10.1016/j.rpth.2023.100154

**Published:** 2023-04-12

**Authors:** Nina Wolska, Meral Celikag, Antonio Virgilio Failla, Anuradha Tarafdar, Thomas Renné, Mauro Torti, Ilaria Canobbio, Giordano Pula

**Affiliations:** 1 Institute for Clinical Chemistry and Laboratory Medicine, University Medical Center Eppendorf, Hamburg, Germany; 2 UK Dementia Research Institute, University College London, London, United Kingdom; 3 Microscopy Imaging Facility, University Medical Center Eppendorf, Hamburg, Germany; 4 Cancer Research Horizons, Babraham Research Campus, Cambridge, United Kingdom; 5 Institute of Biomedical & Clinical Science, University of Exeter Medical School, Exeter, United Kingdom; 6 Irish Centre for Vascular Biology, Royal College of Surgeons in Ireland, Dublin, Ireland; 7 Center for Thrombosis and Hemostasis, Johannes Gutenberg University Medical Center, Mainz, Germany; 8 Department of Biology and Biotechnology, University of Pavia, Pavia, Italy; 9 Biomedical Institute for Multimorbidity, Hull and York Medical School, Hull, United Kingdom

**Keywords:** amyloid peptide β, hypoxia, inflammation, platelet, release

## Abstract

**Background:**

Platelets contain high levels of amyloid β (Aβ) peptides and have been suggested to participate in the deposition of amyloid plaques in Alzheimer’s disease.

**Objectives:**

This study aimed to determine whether human platelets release pathogenic Aβ peptides Aβ_1-42_ and Aβ_1-40_ and to characterize the mechanisms regulating this phenomenon.

**Methods:**

Enzyme-linked immunosorbent assays (ELISAs) and single platelet immunostaining were utilised to study the release and subcellular localisation of Aβ, respectively.

**Results:**

ELISAs revealed that thrombin and the pro-inflammatory molecule lipopolysaccharide (LPS) induce platelet release of both Aβ_1-42_ and Aβ_1-40_. Notably, LPS preferentially induced the release of Aβ_1-42_, which was potentiated by the reduction of oxygen from atmospheric levels to physiological hypoxia. The selective β secretase (BACE) inhibitor LY2886721 showed no effect on the release of either Aβ_1-40_ or Aβ_1-42_ in our ELISA experiments. This suggested a store-and-release mechanism that was confirmed in immunostaining experiments showing co-localisation of cleaved Aβ peptides with platelet alpha granules.

**Conclusion:**

Taken together, our data suggest that human platelets release pathogenic Aβ peptides as a result of a store-and-release mechanism rather than a de novo proteolytic event. Although further studies are required to fully characterize this phenomenon, we suggest the possibility of a role for platelets in the deposition of Aβ peptides and the formation of amyloid plaques. Interestingly, the combination of hypoxia and inflammation that we simulated *in vitro* with reduced oxygen tension and LPS may increase the release of fibrillogenic Aβ_1-42_ and, consequently, exacerbate amyloid plaque deposition in the brain of patients with Alzheimer’s disease.

## Essentials

• Platelets release amyloid β (Aβ) peptides, the main components of brain plaques in patients with dementia.
• Release of Aβ peptides is triggered by platelet activation using different stimuli.
• Availability of oxygen further regulates the release of Aβ peptides by platelets.
• Amyloid β peptides appear to be stored and released by platelet structures called α granules.

## 1 Introduction

Brain hypoperfusion has been reported in Alzheimer’s disease (AD) animal models [1] and patients with AD [2]. The impaired function of the cerebrovascular system leads to brain hypoperfusion and accelerates brain tissue degeneration [3]. Interestingly, high levels of preactivated platelets have been reported in patients with AD [4], and platelet deposits have been shown in the neurovasculature of AD animal models and patients with AD [5]. Growing evidence suggests a role for platelets in the onset and progression of AD [6]. Platelets have been shown to contribute to neuroinflammatory responses [7], neuronal death [6], microthrombotic and ischemic brain complications [8], and cerebral amyloid deposition in AD experimental models and patients with AD [9].

Seminal studies have suggested that circulating platelets express amyloid precursor protein (APP) but failed to convincingly prove the release of pathogenic amyloid β (Aβ) peptides by platelets in physiological or pathological conditions [[10], [11], [12]]. More recently, there have been reports regarding the ability of platelets to release Aβ peptides, although the molecular mechanisms of this phenomenon remain poorly understood [[13], [14], [15]]. Taken together, there is sufficient evidence in the literature supporting a role for platelets as a potential source of Aβ peptides, but several questions remain regarding the events regulating the storage and release of Aβ peptides.

In this study, we have investigated this aspect of platelet biology. In our experiments, the release of Aβ peptides by platelets was induced by hemostatic agonists (ie, thrombin) or pathological stimuli associated with inflammation (ie, lipopolysaccharide [LPS]) and was modulated by oxygen tension. Importantly, although previous studies reported the secretion of Aβ_1-37_ and Aβ_1-40_ peptides [12,13], here we show the release of the more fibrillogenic and pathogenic Aβ_1-42_. This article provides novel evidence of a potential exacerbating effect of inflammation and hypoxia on Aβ peptide release by platelets and potentially on amyloid plaque deposition. In view of our data, recent reports of protection from AD by antiplatelet drugs [16,17] may be explained as a consequence of the inhibitory effects of these drugs on platelet-dependent Aβ peptide release, which would ultimately reduce amyloid plaque deposition and slow down AD progression.

## 2 Methods

### 2.1 Human blood collection and platelet isolation

Human blood was drawn from the median cubital vein of healthy volunteers. Procedures using human blood conformed to the Declaration of Helsinki and underwent local ethics approval (University Clinic Eppendorf Hamburg, UKE). Written informed consent was given by the study participants. Platelet-rich plasma was separated from whole blood by centrifugation (250 × *g*, 17 minutes), and platelets were separated by a second centrifugation step (700 × *g*, 10 minutes) in the presence of prostaglandin E1 (40 ng/mL) and indomethacin (10 μM). Before the experiments, platelets were resuspended in modified Tyrode’s buffer (pH 7.3).

### 2.2 Enzyme-linked immunosorbent assay

Quantikine enzyme-linked immunosorbent assay (ELISA) kits from R&D Systems (DAB142 and DAB140B) were used to quantify human Aβ_1-42_ (aa1-42) and human Aβ_1-40_ (aa1-40) in platelet supernatants (4 × 10^8^ platelets/mL). After treatment with inhibitors and stimuli, platelet supernatants were prepared by removing platelets from suspensions by centrifugation (20,000 × *g*, 1 minute). Where indicated, hypoxia was achieved by equilibrating all solutions for a minimum of 4 hours in 2% O_2_ atmosphere and performing all platelet incubations at 2% O_2_ using a gas treatment chamber (BIO V, Noxygen).

### 2.3 Platelet immunostaining

Human platelets in modified Tyrode’s buffer at a density of 0.5 × 10^7^ platelets/mL were used for these experiments. Coverslips were coated with 0.05-mg/mL fibrinogen. After 30 minutes of incubation, the platelets were fixed with 4% (w/v) paraformaldehyde and permeabilized with 0.5% (v/v) Triton X-100. Immunostaining was performed with anti-Aβ (Abcam, #ab134022), anti–P-selectin (Santa Cruz Biotechnologies, #sc-6941), anti-APP (Abcam, #ab32136), and anti-CD63 antibodies (BD Biosciences, #353030). Fluorescence images were taken using a Zeiss Axio Examiner Z1 with LSM 980 and Airyscan 2 (upright) microscope and analysed for co-localisation using FIJI software (Rasband, W.S., ImageJ, U. S. National Institutes of Health, Bethesda, Maryland, https://imagej.nih.gov/ij/, 1997-2018) and the dedicated plugin “Coloc 2” (source: https://github.com/fiji/Colocalisation_Analysis).

### 2.4 Statistical analysis

ELISA results were compared using 2-way analysis of variance with Bonferroni’s post hoc test. Colocalization results were analyzed using Pearson’s correlation test and the nonparametric Kruskal-Wallis test. Statistical analysis and data presentation were performed using GraphPad Prism version 8.1.0.

## 3 Results

**Figure 1:**
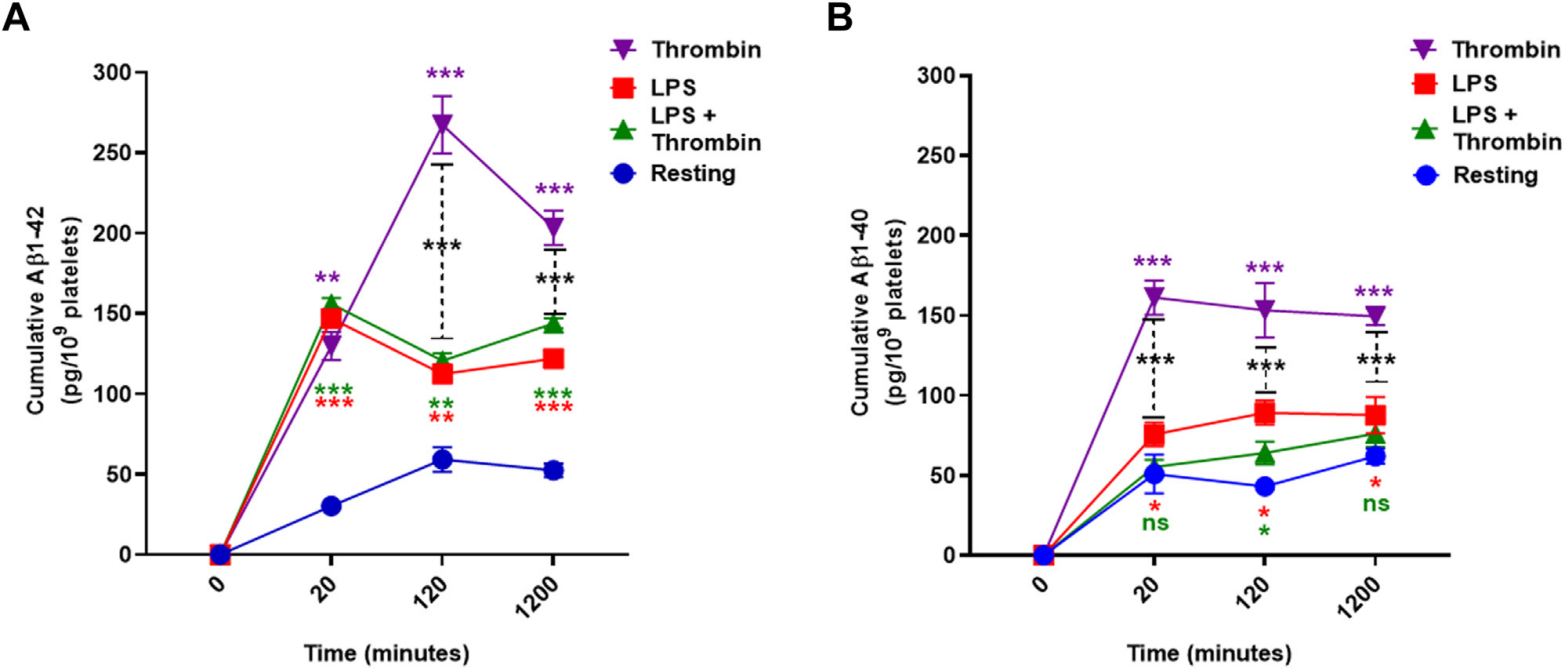
Stimulated release of Aβ_1-42_ (A) and Aβ_1-40_ (B) by human platelets. Isolated human platelets prepared, as described in the Methods section, at a density of 4 × 10^8^/mL were incubated with vehicle solution or 1 unit/mL of human thrombin for 20 minutes, 120 minutes, or 1200 minutes. In parallel or in addition to thrombin, the exposure of platelets to 10 μg/mL of LPS from *Escherichia coli* (NovusBio, #NBP2-25295) was studied. The supernatant was then analyzed by enzyme-linked immunosorbent assay to quantify Aβ_1-42_ and Aβ_1-40_ independently. The statistical analysis was performed by 2-way analysis of variance with the Tukey post hoc test for each data point (n = 4). Colored stars indicate the statistical significance of the relative condition compared with resting platelets at the corresponding time. Black stars indicate the statistical significance of the difference between thrombin and thrombin plus LPS at the different time points. LPS, lipopolysaccharide; ns, nonsignificant. ∗*P* < .05; ∗∗*P* < .01; ∗∗∗*P* < .001.

The release of Aβ_1-40_ and Aβ_1-42_ by human platelets was studied using ELISA. In addition to low release of Aβ_1-42_ and Aβ_1-40_ in resting conditions (<30 pg/mL), platelet stimulation by thrombin significantly increased the release of both Aβ peptides, with different kinetics. The release of Aβ_1-42_ was sustained for at least 120 minutes after stimulation (Figure 1A), whereas the release of Aβ_1-40_ displayed fast kinetics, peaking at 20 minutes after stimulation (Figure 1B). In addition, because the immune receptor Toll-like receptor 4 (TLR4) is expressed in platelets [18] and is involved in AD progression [19], the TLR4/CD14 agonist LPS was tested and displayed the ability to stimulate the release of Aβ_1-42_ (Figure 1A) and, to a lower extent, Aβ_1-40_ by platelets (Figure 1B). As previously shown [20], LPS triggered a moderate increase in P-selectin externalization without integrin activation and did not interfere with the stimulation of these 2 responses by thrombin (data not shown). Surprisingly, the pretreatment with LPS reduced the ability of thrombin to release Aβ_1-42_ and Aβ_1-40_ to levels similar to LPS alone (green line vs purple line in Figure 1A, B).

**Figure 2:**
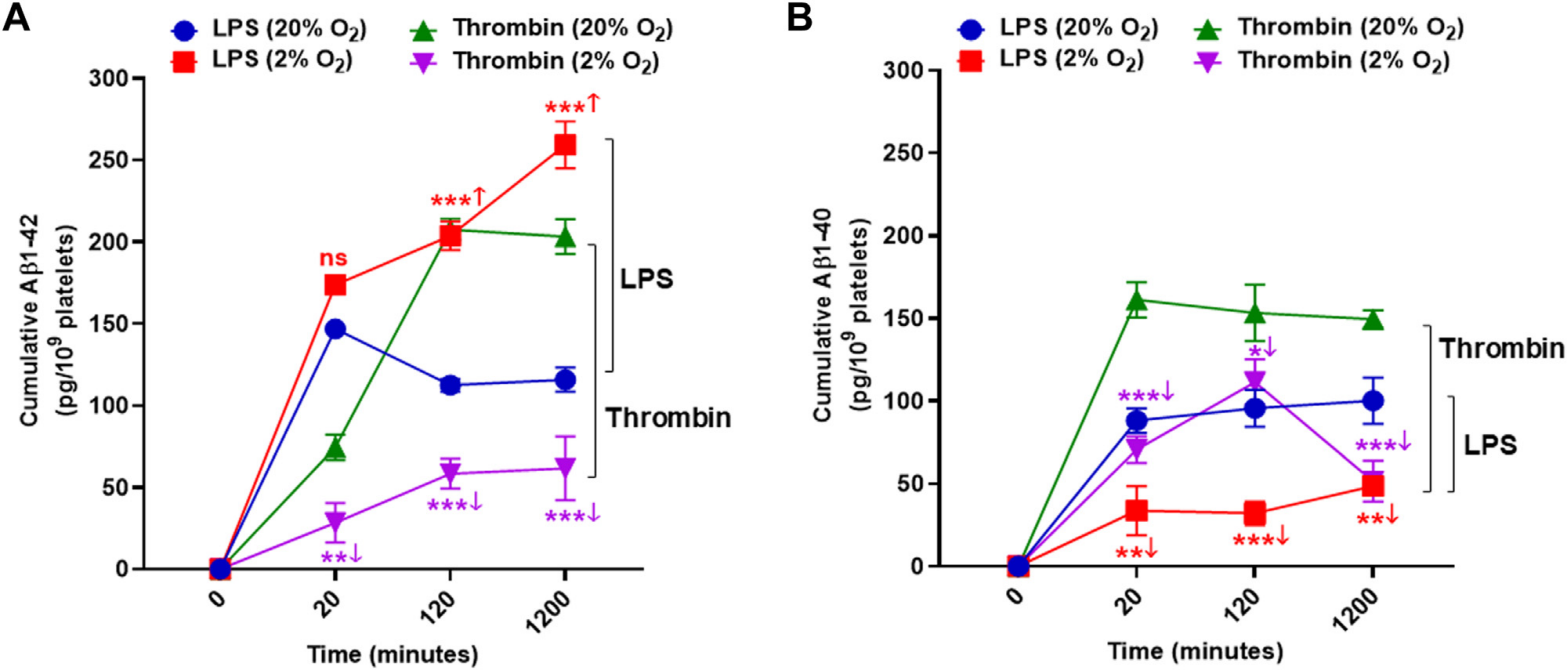
The immune complex Toll-like receptor 4/CD14–dependent release of Aβ_1-42_ by human platelets is potentiated by hypoxia. Isolated human platelets (4 × 10^8^/mL) were stimulated with 1-unit/mL thrombin or 10-μg/mL lipopolysaccharide (LPS) from *Escherichia coli* (NovusBio, #NBP2-25295) in a 20% O_2_ or 2% O_2_ atmosphere. The platelet supernatant after 20, 120, or 1200 minutes of incubation was analyzed by enzyme-linked immunosorbent assay for the presence of Aβ_1-42_ (A) or Aβ_1-40_ (B). The statistical analysis was performed by 2-way analysis of variance with the Tukey post hoc test (n = 4). Each data point was compared to the value obtained in normoxic conditions at the corresponding time from stimulation. The arrow near the statistical significance stars indicates the direction of the hypoxia effect: ↑ indicates increased release and ↓ indicates decreased release. ns, nonsignificant. ∗*P* < .05; ∗∗*P* < .01; ∗∗∗*P* < .001.

Because of the low oxygen tension in the brain microvasculature blood [21] and the reduced neurovascular function that accompanies dementia [22], we assessed the effect of hypoxia on platelet release of Aβ peptides (Figure 2A, B). Interestingly, physiological hypoxia (ie, 2% O_2_ or 15 mm Hg) strongly potentiated the release of Aβ_1-42_ in response to the immune stimulus LPS (Figure 2A), whereas LPS-dependent release of Aβ_1-40_ and thrombin-dependent release of either Aβ_1-42_ or Aβ_1-40_ were reduced in hypoxic conditions (Figure 2B) compared with atmospheric oxygen (ie, 20% O_2_ or 150 mm Hg).

**Figure 3:**
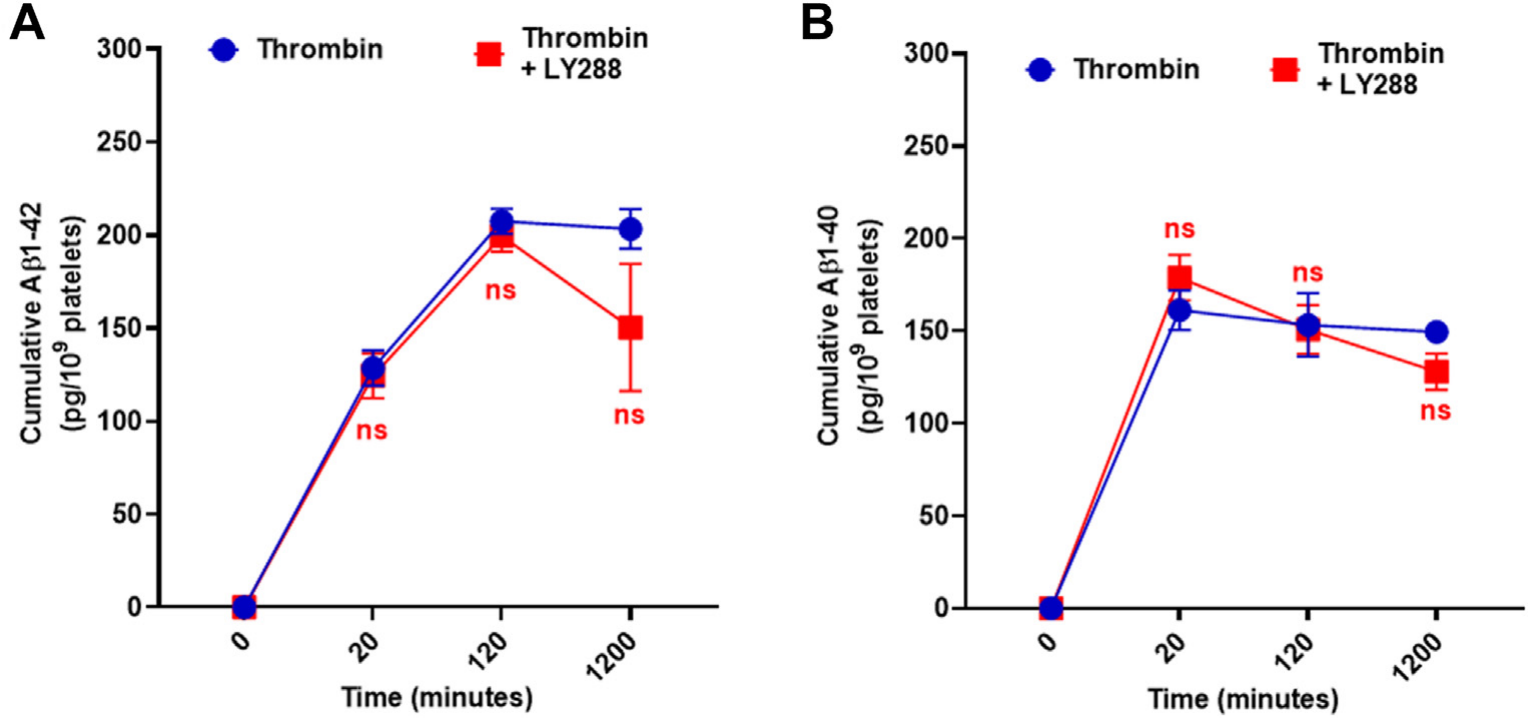
The inhibition of β-secretase by LY2886721 does not affect the release of Aβ_1-42_ by human platelets. Isolated human platelets (4 × 10^8^/mL) were incubated for 30 minutes with 10-μM LY2886721 before stimulation with 1-unit/mL thrombin. The platelet supernatant after 20, 120, or 1200 minutes of incubation was analyzed by enzyme-linked immunosorbent assay for the presence of Aβ_1-42_ (A) or Aβ_1-40_ (B). The statistical analysis was performed by 2-way analysis of variance with the Tukey post hoc test (n = 4). Each data point was compared to the value obtained in the absence of LY2886721 at the corresponding time from stimulation. ns, nonsignificant.

To clarify whether the release of Aβ peptides occurs as a consequence of de novo APP cleavage or a storage-and-release mechanism, we tested the effect of LY2886721, which selectively inhibits β-secretases (ie, the enzymes responsible for the amyloidogenic processing of APP to form Aβ peptides). Despite using a large excess of LY2886721 compared with the expected potency from published data in cellular assays (10 μM in our experiments compared with published IC_50_ values of <20 nM) [23], the release of neither Aβ_1-42_ nor Aβ_1-40_ by human platelets was affected (Figure 3A, B). Because β-secretases are essential for the generation of Aβ peptides in different cell types and cell-free systems [24,25], we suggest that Aβ peptides are already present in the platelets at the moment of the pharmacological treatment.

**Figure 4:**
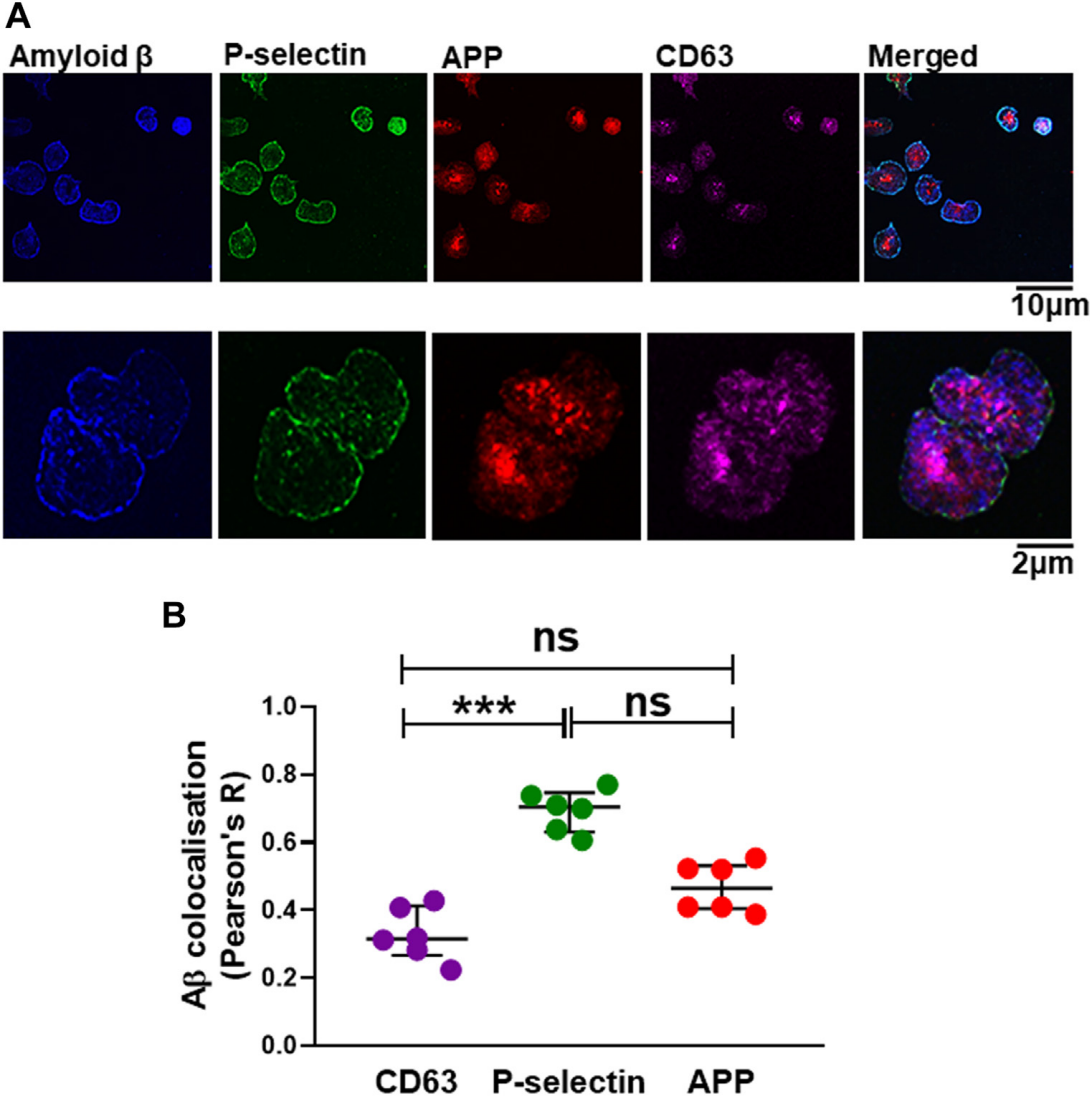
Subcellular distribution of amyloid β (Aβ) peptides in relation to P-selectin, CD63, and amyloid precursor protein (APP). Platelets were allowed to adhere to fibrinogen-coated coverslips for 30 minutes. After fixation and permeabilization, they were immunostained with Aβ-specific, P-selectin–specific, CD63-specific, and APP-specific antibodies. Images were obtained using a Zeiss Axio Examiner Z1 with LSM 980 and Airyscan 2 (upright) microscope with a 63× Plan-APOCHROMAT Oil differential interference contrast objective. Representative pictures are shown in (A). Image analysis was performed using the FIJI software with the dedicated plugin “Coloc 2” (B). Statistical analysis of the colocalization was performed using the Pearson correlation test, and the colocalization was compared between markers using the nonparametric Kruskal-Wallis test. ns, nonsignificant. ∗∗∗*P* < .001; 6 independent experiments.

Immunostaining of human platelets with antibodies selective for Aβ, P-selectin (α granule marker), CD63 (dense granule marker), and APP was used to understand the subcellular localization of Aβ (Figure 4A). Statistical analysis of the costaining revealed high levels of colocalization of Aβ with P-selectin, intermediate levels of colocalization with APP, and low levels of colocalization with CD63 (Figure 4B), which is compatible with Aβ storage in α granules. The intermediate levels of colocalization suggest the possibility of independent storage of APP and Aβ, with at least some α granules storing preferentially one or the other.

## 4 Discussion

The expression of APP in human platelets has previously been suggested [10,11], and the release of Aβ peptides by platelets has been reported, albeit only partially described [[13], [14], [15]]. A solid study of the conditions and the cellular mechanisms leading to Aβ release by platelets is missing. Therefore, the relevance of platelet-driven Aβ secretion for the progression of AD has remained largely elusive.

The most recent and convincing study about this aspect of platelet biology suggested the release of Aβ_1-40_ in response to the activation of protease-activated receptor 1 (ie, the receptor for thrombin) by a selective peptide [15]. On the contrary, we found clear evidence of platelet-dependent release of the more pathogenic form of the peptide upon, that is, Aβ_1-42_. Upon consideration, we found it difficult to support the view that Aβ_1-40_ is the main Aβ peptide released by platelets. In fact, the techniques used in the aforementioned study and similar previous studies [[13], [14], [15]] do not allow appropriate detection of Aβ_1-42_ and cannot distinguish between different Aβ peptides. Among the reasons for the underestimation of Aβ_1-42_ in biological fluids and platelet fractions, there may also be the inherent tendency of this peptide to precipitate and elude quantification in suspension. Our current study benefited from the use of isolated platelets, which eliminates the disturbance by plasma peptides for the detection of platelet-derived Aβ peptides. Moreover, we were able to use an ELISA approach specific for human Aβ detection that distinguishes between Aβ_1-40_ and Aβ_1-42_ and allows side-by-side quantification (ie, in the same samples) of the 2 peptides.

We found that Aβ release was low in the absence of an agonist and that the release of Aβ_1-40_ and Aβ_1-42_ was significantly increased by stimulation of human platelets resuspended at physiological density. The release of Aβ_1-42_ was notably higher than that of Aβ_1-40_ (ie, ∼150 pg/mL for Aβ_1-40_ and ∼250 pg/mL for Aβ_1-42_). The higher level reached by Aβ_1-42_ seems to be due to the prolonged kinetics for the release of this peptide, which continues for at least 120 minutes after activation, rather than a higher release rate (which is similar between Aβ_1-40_ and Aβ_1-42_ in the first 20 minutes after stimulation). This may imply a different mechanism of release for the 2 Aβ peptides, which is an unexpected and highly intriguing finding requiring further study.

Platelets are known responders to infection through the TLR4/CD14 immune complex [18]. Although there is no previous information regarding the effect of the TLR4/CD14 agonist LPS on platelet release of Aβ peptides, previous studies have reported the potentiation of platelet hemostatic responses by LPS [20,26]. In our experiments, the activation of TLR4/CD14 by LPS induced the release of Aβ peptides, albeit at a lower rate than that with thrombin. Our data suggest that platelets can release Aβ peptides independent of canonical hemostatic stimuli. Importantly, the release of Aβ1-42 peptides by LPS was significantly increased by hypoxia. Therefore, infection and inflammatory conditions may trigger platelet release of considerable amounts of pathogenic Aβ peptides if combined with hypoperfusion (which is a condition commonly associated with AD) [2,3]. Further research is required to ascertain the potential relevance of this observation for human health and especially for AD, for which infections [27,28] and/or inflammatory conditions [29] are recognized risk factors. Noticeably, there was no significant difference between the release of Aβ_1-42_ and Aβ_1-40_ induced by LPS either alone or in the presence of thrombin. We can therefore hypothesize that LPS triggers a release mechanism that “overrules” the effect of thrombin. Further mechanistic studies are needed to test this hypothesis.

Our negative data with the β-secretase inhibitor LY2886721 and the immunostaining results suggest that Aβ peptides exist in platelets in their cleaved form (ie, not only as part of APP). Although the possibility of unknown alternative proteases participating in the generation of Aβ peptides in platelets cannot be completely excluded at this point (eg, calpain [12]), it seems plausible that platelets already store β-secretase–dependent Aβ peptides at the time of isolation from blood. Aβ peptides colocalize with P-selectin but not CD63, suggesting their storage in α granules. Our costaining experiments showed consistent but incomplete colocalization of Aβ peptides and APP (Pearson r value, ≈0.5). This may suggest that different Aβ peptides are stored in different α granule subpopulations. The existence of different subpopulations of α granules characterized by different contents has been described previously [30]. The existence of subpopulations of α granules with different contents of Aβ_1-42_ and Aβ_1-40_ peptides may also explain the difference in the kinetics and modulation of the release of these alternative forms of Aβ peptides. Further studies are required to confirm the possibility that APP and Aβ peptides are distributed in different α granules and determine whether there is a correlation between the distribution of these proteins with other cargo proteins of the α granules.

Taken together, this research report presents a strong case for further investigations of the mechanisms of storage and release of Aβ peptides by platelets. These data may improve our understanding of AD and promote the discovery of a treatment to reduce or arrest its progression.
